# Deuterium Metabolic Imaging—Rediscovery of a Spectroscopic Tool

**DOI:** 10.3390/metabo11090570

**Published:** 2021-08-25

**Authors:** Ilona Polvoy, Hecong Qin, Robert R. Flavell, Jeremy Gordon, Pavithra Viswanath, Renuka Sriram, Michael A. Ohliger, David M. Wilson

**Affiliations:** 1 Department of Radiology and Biomedical Imaging, University of California, 185 Berry St., San Francisco, CA 94158, USA; Ilona.Polvoy@ucsf.edu (I.P.); Hecong.Qin@ucsf.edu (H.Q.); Robert.Flavell@ucsf.edu (R.R.F.); Jeremy.Gordon@ucsf.edu (J.G.); Pavithra.Viswanath@ucsf.edu (P.V.); Renuka.Sriram@ucsf.edu (R.S.); Michael.Ohliger@ucsf.edu (M.A.O.); 2 Department of Radiology, Zuckerberg San Francisco General Hospital, San Francisco, CA 94110, USA; 3 Department of Radiology and Biomedical Imaging, University of California, 505 Parnassus Ave, San Francisco, CA 94143, USA

**Keywords:** deuterium, imaging, spectroscopy, magnetic resonance, metabolism

## Abstract

The growing demand for metabolism-specific imaging techniques has rekindled interest in Deuterium (^2^H) Metabolic Imaging (DMI), a robust method based on administration of a substrate (glucose, acetate, fumarate, etc.) labeled with the stable isotope of hydrogen and the observation of its metabolic fate in three-dimensions. This technique allows the investigation of multiple metabolic processes in both healthy and diseased states. Despite its low natural abundance, the short relaxation time of deuterium allows for rapid radiofrequency (RF) pulses without saturation and efficient image acquisition. In this review, we provide a comprehensive picture of the evolution of DMI over the course of recent decades, with a special focus on its potential clinical applications.

## 1. Introduction

Magnetic resonance spectroscopy (MRS) is a powerful method for studying metabolism based on the conversion and/or incorporation of a substrate labeled with non-radioactive nuclei such as ^2^H, ^13^C, and ^15^N [1,2,3]. While the interest in non-radioactive compounds has somewhat subsided in recent years, mostly due to an increased use of radiotracers, Deuterium (^2^H) Metabolic Imaging (DMI) has been recently regaining popularity [4]. Given the low natural abundance of ^2^H (0.01%) [1], the detection of an administered deuterium-enriched substrate and its metabolic products is possible with minimal background signal. Moreover, when DMI is performed alongside routine ^1^H imaging for anatomical localization, the technique provides insight into the spatial biochemistry of living tissue [5,6,7]. DMI is therefore analogous to other techniques that depend on ingestion or infusion of a metabolite modified for NMR, although as we shall see several of the most promising ^2^H probes are also closely related to tools used in positron emission tomography (PET) or chemical exchange saturation transfer (CEST) imaging.

The study of deuterated compounds dates to 1932, when Urey et al. revolutionized the field of inorganic chemistry with the discovery of ^2^H_,_ a heavy isotope of hydrogen [8]. Shortly thereafter, Lewis and colleagues succeeded in enriching water with ^2^H, and from that point on, enrichment of other nuclei such as ^15^N, ^19^O, and ^13^C, became frequent in scientific investigations [9]. Following this discovery, Rittenberg and Schoenheimer published numerous reports in which they administrated ^2^H-containing fat to mice, and identified and described the principles of fatty acid metabolism as well as the synthesis of cholesterol [10,11,12,13,14,15]. This work promoted the use of ^15^N for the study of amino acids [16] and the realization that amino acids labeled with ^2^H retain their label post hydrolysis, thus enabling tracking of protein metabolism [17,18]. This investigation of fatty acid metabolism inspired the study of biological membranes in the early 1970s. Oldfield et al. used specifically deuterated lipids to study the hydrocarbon chain mobility of membrane systems [19] and later, incorporated large amounts of deuterated fatty acids into the membrane of *Acholeplasma laidlawii B* and showed that both liquid crystalline and gel states can be present at the same temperature [20,21]. Following these studies, Arvidson et al. labeled lecithin and sphingomyelin with [Me-^2^H] choline in an in vivo model of rat-liver mitochondria in order to investigate lipid structure and lipid-protein interactions [22]. This pioneering work laid the groundwork for future use of ^2^H NMR to study the dynamic nature of biological membranes [20,23]. Moreover, deuterium membrane research was extended beyond phospholipids to membrane proteins, including pumps [24], channels [25] and receptors [26]. ^2^H NMR provided important insights into the synthesis and metabolism of those key membrane components, and the interactions between them [27].

Another significant milestone related to ^2^H in the field of biochemistry was the discovery of the kinetic isotopic effect, and its use in probing reaction mechanisms. In 1952, Ramsay et al. [28] identified that isotopes differ in their vibrational frequencies and centrifugal stretching, meaning that a bond containing heavier isotopes will require larger amounts of energy to reach the activation energy of a reaction. Unlike other isotopic pairs (i.e., ^12^C versus ^13^C), the difference in mass between hydrogen and deuterium is a factor of 2, leading to a significantly lower zero-point energy of the deuterium-carbon bond compared to the proton-carbon bond. This leads to higher bond stability, and thus increased time to break C-D bonds. The kinetic isotope effect thus allows determination of rate limiting steps and reaction transition states, [29] thus improving understanding of reaction mechanisms [30]. Moreover, recent studies have used the kinetic isotopic effect by creating deuterium-containing drugs that that are less metabolically active thus optimizing their stability [31,32]. 

In this review, we focus on recent advances in ^2^H-based imaging for gaining unique insights into metabolism in real-time. Although ^2^H has been used to study metabolism for many years, its use as a practical imaging tool has been delayed. Historically, this was likely due to the increased use of radioactive isotopes which were more affordable, easier to prepare and administer and had higher sensitivity [4]. In addition, the high abundance (99.98%) and sensitivity (highest gyromagnetic ratio) of the hydrogen nucleus has led to rapid evolution of tools for ^1^H NMR including many spectroscopic methods that are now integrated into routine clinical practice, especially for the characterization of prostate tumors [33,34], brain tumors [35,36,37], and various other metabolic derangements [38,39,40]. As far as other metabolic imaging tools, PET has also been extraordinarily successful especially with the rapid proliferation of metabolite-derived tracers and the expansion of dual modality scanners (i.e., PET-CT and PET-MR) [41,42,43,44]. Finally, imaging of other stable isotopes (i.e., ^13^C) has benefitted from dynamic nuclear polarization (DNP) [45,46], where the nuclear polarization (and therefore the MR signal) is transiently increased by several orders of magnitude prior to injection. 

The main arguments for further developing DMI metabolic imaging tools, to complement these methods are as follows: (1) ^1^H MRS is only able to detect abundant high concentration metabolites in situ at steady state; (2) PET uses ionizing radiation, and the most frequently studied probes (e.g., fluorine-derived) are not chemically identical to endogenous substrates; (3) the technical requirements of hyperpolarized ^13^C (and ^15^N) MRS limit their applications to a select number of suitable metabolites. In contrast DMI allows the use of ^2^H-enriched metabolites with high homology to endogenous molecules, with potentially more flexible dosing and scan timing. Based on reported ^2^H-enriched metabolites studied via DMI, this field is certainly in its nascency with accelerating interest in recent years.

## 2. DMI-Companion Metabolic Imaging Tools

In this review, we highlight deuterium magnetic resonance methods that rely on the metabolism of an administered substrate, such as D-[6,6′-^2^H_2_] glucose or [2,3-^2^H_2_] fumarate. This focus is driven by the recent interest in D-[6,6′-^2^H_2_] glucose, which has been applied to study normal brain glucose metabolism [47] as well as to patients with glioblastoma (brain tumors) [1]. Given this development of DMI techniques for glucose, one relevant technology is positron emission tomography (PET) using D-[2-^18^F]-2-deoxyglucose (FDG). FDG is frequently applied to patients with cancer in clinical practice, highly sensitive to small tumors and compatible with full-body imaging (PET-CT or PET-MRI) [48,49,50]. PET using FDG relies on the enhanced glucose transport in cancer and other lesions. FDG is transported into tumors using GLUT 1,3,4 transporters and phosphorylated at the 6-position by hexokinase. The resulting adduct cannot undergo isomerization to form the corresponding ^18^F fructose sugar and is thus “trapped” with the PET signals obtained fundamentally reflecting enhanced glucose uptake and phosphorylation [51,52]. In contrast, D-[6,6′-^2^H_2_] glucose for DMI is transported in an analogous manner, but undergoes further downstream metabolism and can detected via high concentration metabolites such as lactate (in tumor, or stroke) or glutamate/glutamine in normal and pathological tissues. Recent work has highlighted the way DMI can be performed via 3-dimensional chemical shift imaging in both animal (2 × 2 × 2 mm^3^) and human (20 × 20 × 20 mm^3^) applications, providing spatial resolution comparable to PET and ^1^H spectroscopic imaging [1].

Another related technology is CEST imaging, specifically “glucoCEST”, a method used to image glucose uptake by glycolytic tumors in vivo [53], recently described in patients with gliomas [54] as well as head and neck tumors [55]. Similarly to DMI, this method interrogates increased glycolysis without relying on radiolabeling, offering the prospect of a faster, safer and cheaper process compared to [^18^F]-FDG PET [53,56]. CEST is an MRI contrast enhancement technique which is based on a proton exchange between the compound of interest (endogenous molecules such as amides, glucose or glutamate and exogenous molecules such as diamagnetic molecules, liposomes, etc.) and water. This is accomplished by applying a radiofrequency pulse to a specific compound in order to reach its saturation point. At this point, the compound is able to transfer its proton signals to the water pool, creating a visible contrast allowing the indirect detection of the compound of interest [57]. Unlike CEST, DMI allows for the direct observations of those compounds; however, the sensitivity and spatial resolution of CEST is reportedly higher [58]. 

From a technical perspective, DMI is also closely related to other spectroscopy methods employing stable isotopes, such as hyperpolarized ^13^C MRI [1]. In this method, a long T_1_ nucleus is ^13^C-enriched, and polarized at low temperature in the presence of an organic radical and microwave energy. Following dissolution, the molecule can be applied to a variety of biologic systems including human patients. The most commonly used probe is hyperpolarized [1-^13^C]pyruvate, which is converted to metabolic products including D-[1-^13^C]lactate that correlate with various disease states [59,60]. This method provides superior signal to noise ratio as well as spatial resolution compared to DMI. Moreover, its chemical shift spread enables an easier signal resolution. However, the longer acquisition time of DMI allows to observe not only the lactate but also Glx (glutamine and glutamate) production, thus creating metabolic map for both oxidative and non-oxidative pathways of glucose metabolism [61]. A variety of other analogous methods have been used relying on bioconversion of an administered probe including hyperpolarized ^15^N MRI, as well as studies at thermal equilibrium using ^13^C, ^15^N, ^19^F, and ^31^P [62,63,64,65,66]. 

Of course, no discussion of spectroscopy is complete without ^1^H MRS, the technique actually used in patient scans with applicability to a large number of diseases. ^1^H MRS can detect highly abundant, NMR-observable metabolites at steady state in situ, meaning that it does not require prior injection of an imaging agent [67]. Depending on the nucleus, especially its chemical shift and spin-spin (T_2_) relaxation, and the spectroscopic parameters used (in particular the echo time, TE), several metabolites modulated in disease states can be detected. In clinical practice, long TE ^1^H MRS can detect steady-state choline, creatine, N-acetyl aspartic acid (NAA) and lactate. Short TE ^1^H MRS can also detect shorter T_2_ metabolites such as glutamine and glutamate. Recent work has also highlighted that spectral editing and 2-dimensional ^1^H MRS [68] techniques can be used to “uncover” steady-state metabolites of high interest, for example, 2-HG, an oncometabolite present at high concentrations in patients with isocitrate dehydrogenase mutant (IDHm) tumors [69,70]. Furthermore, a recent study found that spectral changes after administration of deuterated compounds, such as [6,6′-^2^H_2_] glucose and [2,2,2′-^2^H_3_] acetate, could be detected by ^1^H MRS, showing the potential synergy between ^1^H MRS and deuterium-enriched methods [71]. 

## 3. DMI Methodology

DMI is based on chemical shift detection of deuterated molecules. The natural abundance of ^2^H is low (0.0115%), resulting in approximately 10 mM concentration signal, an extremely low signal relative to the one arising from water and corresponds to 110 M protons [1,72]. However, this low natural abundance is considered a major advantage regarding detection of an exogenous ^2^H-enriched substrate and its products above background as well as serving as a quantitative internal reference. As highlighted by the de Graaf work using D-[6,6′-^2^H_2_] glucose, the enriched substrate can be delivered either intravenously (rats) or orally (humans) with detection of lactate, and [^2^H] glutamine/glutamate via ^2^H-MRS after approximately an hour [1]. This work also rendered 3-dimensional DMI maps of ^2^H-enriched metabolites using chemical shift imaging. Brain and liver studies in animals used a dual-tuned ^2^H/^1^H coil design whereby the ^2^H coil was mounted inside of the ^1^H coil. Human studies also used a dedicated ^2^H coil, with other recent work highlighting the way standard ^1^H RF coils could also be used to detect ^2^H metabolites indirectly via the reduction of normal signals [71]. For a full description of the techniques used for human ^2^H DMI, please refer reference [1]. Observations from this and other reports support the following general considerations regarding application of DMI to metabolic imaging:

*Sensitivity:* Factors impacting sensitivity of DMI include the ^2^H gyromagnetic ratio, magnetic moment, linewidth, J-coupling, and T_1_/T_2_ relaxation times. The gyromagnetic ratio g of ^2^H is approximately 6.5 times lower than that of ^1^H, a feature that is partially offset by its larger magnetic moment which is created by a spin quantum number of 1 compared to the ½ of ^1^H [1]. Sensitivity to ^2^H is also negatively impacted by short spin-spin relaxation times, broader linewidths, and J-coupling. Despite these effects on sensitivity, the nuclear spin-lattice relaxation times of ^2^H are short (approximately 10-fold shorter than the relaxation times of ^1^H), allowing more rapid radiofrequency (RF) pulses without saturation, markedly improving scan efficiency for a given metabolite concentration. An additional factor dramatically affecting the detection of ^2^H is the field used since SNR for ^2^H has approximately a quadratic relationship with the static magnetic field. Given the short T2 and broad linewidths for ^2^H compounds, 2D or 3D chemical shift imaging (CSI) methods have been the pulse sequence of choice for DMI [1,6].

While these technical considerations are essential for any prospective ^2^H probe, compelling in vivo biochemistry is most important. If the goal is metabolism of substrate to product (the focus of this review), this conversion needs (1) to occur at the appropriate time-scale post injection; (2) to produce a product with distinct (or resolvable) chemical shift; (3) to produce a product that is NMR-detectable; and (4) to produce a product with a sufficiently high concentration. For applications in patients, the metabolic pathway interrogated must be relevant to the detection and/or treatment of disease.

*Chemical shifts interrogated:* The chemical shifts of ^2^H nuclei follow those of their ^1^H counterparts and are thus easily predicted using a metabolomics database [73], commercially available ^1^H spectra, or routine 1D NMR of proton-containing compounds. The ^2^H spectrum of biologically relevant enriched metabolites is somewhat compressed (similar to ^1^H-MRS), which might create difficulty in separating the different resonances. An example of that is the unsuccessful attempt made by Feyter et al. to resolve D-[6,6′-^2^H_2_] glucose from glycogen in the ^2^H spectrum due to overlap of these resonances [74]. The contribution of natural abundance HDO (about 10 mM) represents a potential concern if the intent is to detect ^2^H substrates or metabolites with similar chemical shifts (around 4.7 ppm). Because of this contribution and the relatively broad spectral linewidths of water in ^2^H NMR (about 11.8 Hz and 11.7 Hz for 4 T and 7 T, respectively) [6], it is generally advantageous to detect nuclei with a larger chemical shift difference with respect to water, for example D-[^2^H] lactate (1.3 ppm). Ideally, although numerous reports suggest that water suppression is not required for deuterium magnetic resonance, the observed metabolite should be >1 ppm away from water and be resolved from the administered probe. A related difficulty is detecting and quantifying two distinct metabolite chemical shifts, based again on broad linewidths and SNR. 

*Safety:* Deuterated molecules have been studied in humans for many years, especially water itself (D_2_O) [75,76,77,78,79]. Deuterium was shown to be lethal when it enriches the total body water by 30-40%, while side effects appear at 10–20% [80]. Of note, the usual dose range administrated is subjects, will be equivalent to approximately 0.5–1.5% rise in the enriched body water, significantly below the toxicity threshold. For example, in the De Feyter study, the D-[6,6′-^2^H_2_] glucose dose administered to human subjects was 0.75 g/kg, with a maximum of 60 g, increasing the enriched TBW by merely 0.01% for a 80 kg human subject [1,4]. Moreover, this represented a significantly decreased dose versus other human applications reported. Increasingly, other applications are using deuterated (drugs) molecules and solvents most notably hyperpolarized ^13^C NMR to increase T_1_ relaxation times [81]. Based on all available data, it appears likely that ^2^H will not represent a major safety concern in various patient MRI applications [82].

## 4. Probes for DMI

Only a few deuterium-enriched probes targeting metabolic pathways have been reported, in contrast to ^13^C/^15^N substrates for hyperpolarized MRI [83], or even ^13^C and ^19^F molecules for NMR/MRI at thermal equilibrium [84,85]. At this stage the basic requirements appear to relate to biochemical feasibility (especially, uptake and conversion on the time scale of the experiment), and chemical shift of the detected metabolite. Examples of ^2^H-enriched probes that have been studied in vitro or in vivo include glucose, acetate, choline, fumarate, and water (D_2_O) itself (Table 1).

**Figure 1:**
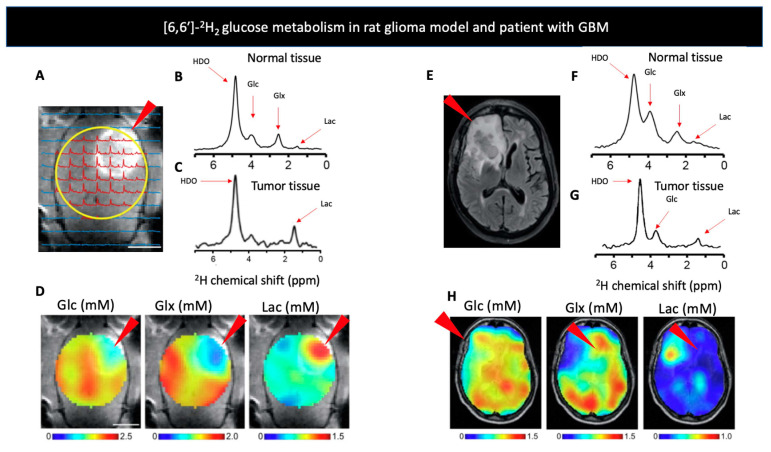
(**A**) Contrast-enhanced T_1_-weighted MRI image of a rat glioma model. The arrow indicates increased signal from the tumor region, with the ^1^H image overlaid with the localized ^2^H MR spectra. The yellow circle indicates the position of surface coil. (**B**,**C**) show deuterium NMR spectra acquired after infusion of [6,6′-^2^H_2_] glucose (Glc) in a normal (**B**,**C**) tumor bearing rats’ brain. Red arrows indicate the chemical shifts of HDO, Glc, glutamate and glutamine (Glx), and [6,6′]-^2^H_2_ lactate (Lac). (**D**) Metabolic uptake maps from a rat glioma model produced after injection of Glc. Arrows indicate increased uptake of Glx and lactate around the tumor region. (**E**) T_2_-weighted fluid-attenuated inversion recovery MR image of a patient with glioblastoma multiforme (GBM), arrow indicates increased signal from tumor region. (**F**,**G**) show deuterium NMR spectra acquired after oral administration of Glc in a healthy volunteer (**F**,**G**) patient diagnosed with GBM, red arrows indicate the chemical shifts of HDO, Glc, Glx, and Lac. (**H**) Metabolic uptake maps from a patient diagnosed with GBM produced after oral administration of Glc. Arrows indicate increased uptake of Glx and lactate around the tumor region. Adapted from De Feyter et al. 2018 (non-commercial 4.0 international license, CC BY-NC 4.0).

*D-[6,6′-^2^H_2_] glucose:* Our discussion begins with D-[6,6′-^2^H_2_] glucose, which has recently been studied in patients and whose uptake and conversion is analogous FDG used in PET- the current gold standard for metabolic imaging in cancer patients. Older studies using D-[6,6′-^2^H_2_] glucose have analyzed bacterial metabolism [86,87,88], retinal metabolism [89,90,91], glycogen synthesis [92], and red blood cell metabolism [93]. Recent studies have targeted the Warburg effect, the increased anaerobic glycolysis in the presence of sufficient oxygen levels that is characteristic of cancer cells [94]. While, [^18^F]-FDG PET, the most common tool targeting metabolism, transport of FDG serves as a surrogate for glycolysis [95], DMI can follow the metabolism of glucose itself. In the 2018 De Feyter study, DMI with [6,6′-^2^H_2_] glucose was used to map the glycolysis and oxidative phosphorylation in both a rat glioma model and 2 patients with glioblastoma to show a higher lactate/Glx ratio in the disease areas (Figure 1), as well as the response to treatment with dichloroacetate. Moreover, they showed the storage of [6,6′-^2^H_2_] glucose as glycogen in the liver of both rats and humans [1]. However, a more recent attempt to visualize liver glycogen storage by providing mice with [6,6′-^2^H_2_] glucose in drinking water did not achieve the desired ^2^H-labeled glycogen signal due to a very short T_2_ (<2 ms) [74]. Kreis et al. [3] took the use of D-[6,6′-^2^H_2_] glucose even further and presented a method to quantitate the tumor glucose flux while using 3D accelerated chemical shift images of the tumor before and after chemotherapy in a murine lymphoma model. Later, Rich et al. [71] applied a novel approach called quantitative exchanged-label turnover MRS (qMRS), in which they infused healthy and glioblastoma bearing rats with deuterated compounds of glucose and acetate, and imaged them with ^1^H MRS. This allowed detection of changes in the ^1^H MRS spectra, following ^2^H incorporation. This method thus provides improved spectral resolution and enables not only the tracking of metabolite production but also their quantification without the need for specialized ^2^H coils [71]. Recently, Markovic et al. used DMI to demonstrate the increased anaerobic glycolysis in preeclamptic rodents compared to control. They showed not only the increased production of [3,3′-^2^H_2_] lactate concentration in the placenta and fetal organs of those animals, but also a notable increase in the heavy water concentration, after injecting [6,6′-^2^H_2_] glucose. Those results further support the claim that preeclampsia is a hypoxic condition [96]. Furthermore, the same group used DMI to monitor tumor metabolic activity of 2 distinct pancreatic ductal adenocarcinoma mice models, and showed an increase in [3,3′-^2^H_2_] lactate regardless of the model used. This method, however, showed limitations when attempting to detect small tumors, less than 5mm in size [97].

Several studies have also analyzed the metabolism of glucose in the normal and ischemic brain, and adipose tissue. Lu et al. used ^2^H MRS to assess the rate of glucose consumption and TCA cycle flux in a rat’s brain under normal conditions as well as under morphine and 2% isoflurane and showed significantly higher rates of neuronal activity in morphine treated rats compared to the anesthetized ones [47]. Straathof et al. showed glucose metabolism after ischemic stroke in rats by presenting a significant increase in lactate formation in the diseased animals [98]. Furthermore, a recent publication compered DMI and HP [1-^13^C] pyruvate in imaging of cerebral metabolism, showing that they are both feasible methods at 4.7 T [61]. An alternate approach, described recently in Mahar et al., was to assess cerebral glucose metabolism by administering [^2^H_7_] glucose by looking at the HDO as the metabolic product, bypassing the need for lower resonance products such as lactate and Glx and showing that HDO can indeed serve as a biomarker of [^2^H_7_] glucose, lactate, and Glx metabolism [99]. Riis-Vestergaard et al. reported the effect of temperature on metabolically active brown adipose tissue. This was achieved by showing a higher [6,6′-^2^H_2_] glucose signal along with a fast decay and without a significant difference in lactate levels, in rats housed in cold conditions compared to those in neutral temperatures, a result comparable to that seen previously for [^18^F]-FDG PET [100].

*D_2_O:* Water is essential to numerous biosynthetic pathways, including protein, lipid and nucleic acid synthesis. Administering heavy water enables its incorporation into cellular pools, followed by its use in various reactions, such as hydrolysis or dehydration, providing the detection of multiple different metabolites [101]. The use of heavy water (D_2_O) as an NMR probe dates back to the 1930s, and was heavily used in the field of biochemistry [79]. This was accomplished either by labeling molecules with D_2_O and observing the reactions’ sequence or by measuring the incorporation of the heavy water into different products after a direct administration of D_2_O to a subject [102]. Both of those methods were used by Rittenberg and Schoenheimer in their study of lipid metabolism in order to calculate rates of various reactions such as lipid turnover, biosynthesis, incorporation into fatty tissue and so on [102,103]. In 1986, Brereton et al. administrated 10% D_2_O in drinking water to mice in order to study the deuterium turnover in water and lipids [75]. The same group later investigated the fat use rates in both obese and diabetic mice models with significantly lower rates in the former but not in the latter, compared to controls [104]. Another interesting application of D_2_O was introduced by Kim and Ackerman in the late 1980s, when DMI was used to measure blood flow and tissue perfusion by showing the clearance of D_2_O from the liver of mice [76]. Following this study, the determination of cerebral [105] and myocardial [106] blood-flow rates and perfusion were soon achieved. This was done by either exposing the animals to D_2_O in drinking water [105] or injecting a bolus of D_2_O directly into the organ in question [76,106]. Soon thereafter, the assessment of tissue perfusion was applied to several disease sates. Ackerman et al. evaluated the tumor blood flow (TBF) in a in vivo RIF-1 tumor model [107,108]. Later, this method was used to assess tumors’ response to treatment [109] as well as to examine the distribution of the tracer inside the tumor [110,111]. Stroke was another important target, with Kito et al. showing a significant change in the ^2^H NMR signal intensity in areas of decreased perfusion following brain infarction in a rabbit model [112]. Obata, T. et al. used intraperitoneal injection of deuterated saline to the visualize the dynamic changes of water in rats’ eyes [113]. In addition, the administration of D_2_O was used extensively in the past decade to assess the proliferation of cells by evaluating the incorporation of deuterium into the DNA through the de novo nucleoside synthesis pathway, in both human and animal models [77,114,115]. This provoked the recent work done by Buxbaum et al. that used deuterium MRI to identify organs infiltrated by alloreactive T cells in graft-versus-host disease [78]. Moreover, this group has showed that continuous labeling of D_2_O can be a valuable measurement of tumor cholesterol metabolism in mice, and while this work has not been peer reviewed at the time of this writing, it represents a promising direction [116].

*[^2^H_3_] acetate:* Enriched acetate derivatives have always been of interest due to the ability of exogenous acetate to be converted into acetyl-CoA, the product of pyruvate decarboxylation that is readily incorporated into intermediates of the tricarboxylic acid (TCA) cycle as well as other molecules [1,117]. Furthermore, it has been previously shown that brain tumors, both those originates from the brain as well as metastases from a broad range of cellular origins, show increase uptake of acetate. This is especially interesting given that most tumors are unable to metabolize acetate in their source tumor and must undergo specific genetic adaptations to the brain microenvironment to do so. In 2018, De Feyter et al. used this fact in order to reveal differences in metabolism of [^2^H_3_] acetate between normal brain and tumor tissue in a rat glioma model. It was found that tumor samples had significantly greater levels of acetate and lower levels of acetate oxidation compared to healthy brains. However, it remains unclear whether the increase in monocarboxylic transporter is what facilitate the increase in acetate uptake or the compromised blood-brain barrier that is also characteristic of this tumor model [1]. Following this work, Rich et al. manage to provide insight about both steady-state and metabolic rates for several key metabolic players using qMRS [71].

*[^2^H_9_] choline chloride:* Choline is a vital dietary nutrient with important functions in both human and animal bodies. It is mostly found in phospholipids, but it also has other essential roles such as methyl donor and as a precursor to acetylcholine, an important neurotransmitter [118]. An experiment conducted by Eng et al. analyzed the distribution and metabolic fate of methyl groups in a rabbit kidney model, following an infusion of [^2^H_9_] choline. They showed high levels of [^2^H_9_] betaine, an important [^2^H_9_] choline chloride metabolite, in the cortex of the kidney, and to a lesser degree in the inner medulla. This was reinforced further following administration of diuretics [119]. Later, Katz-Brull et al. examined the biochemical mechanism behind the observation that malignant breast cells have higher phosphocholine levels compared to healthy tissues. This was achieved by administering [^2^H_9_] choline to nude mice inoculated with MCF7 cells and identifying the increased cell uptake, accumulation and choline phosphorylation in tumor cells [120]. Currently, choline metabolism is being investigated in a rat glioblastoma model [121]. Recently, [^2^H_9_] choline was used in single voxel MRS of the liver to measure the choline concentration in healthy volunteers following randomly assigned diets of variable choline levels as well as a choline bolus 72 h prior to scanning. However, no significant correlation between the choline intake and the choline liver concentration was noted [122].

**Figure 2:**
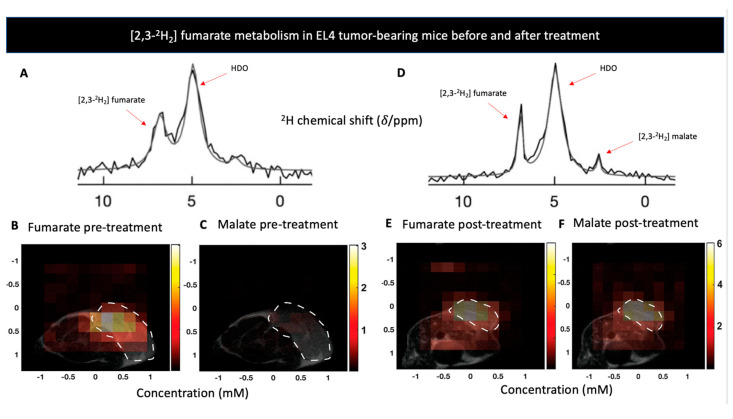
(**A**) The sum of 12 deuterium NMR spectra during 60 min of [2,3-^2^H_2_] fumarate infusion to a lymphoma-bearing mice (EL4) prior to treatment with Etoposide. (**B**,**C**) show metabolite concentration maps of [2,3-^2^H_2_] furmarate and [2,3-^2^H_2_] malate prior to treatment with Etoposide in the EL4 model. Tumor locations are outlined by the white dotted lines. (**D**) The sum of 12 deuterium NMR spectra during 60 min of [2,3-^2^H_2_] furmarate infusion to EL4 tumor model after a treatment with Etoposide, shows production of [2,3-^2^H_2_] malate. (**E**,**F**) show metabolite concentration maps of [2,3-^2^H_2_] furmarate and [2,3-^2^H_2_] malate 48 h after to treatment with Etoposide, displaying an increase in malate concentration. Adapted from Hesse et al. 2021 (Creative Commons Attribution License 4.0, CC BY).

*[2,3-^2^H_2_] fumarate:* Fumarate, a product of succinate oxidation in the TCA cycle, can be hydrated by the enzyme fumarase, to produce malate. This conversion was recently exploited by Hesse et al. to detect tumor cell death [123]. They relied on previous studies that used [1,4-^13^C_2_] fumarate conversion to [1,4-^13^C_2_] malate to study tumor cell necrosis following chemotherapy with the premises that the impaired membrane following the treatment will allow higher conversion rate of fumarate to malate [124,125]. In this study [123], the authors used dynamic 3D deuterium MRS in three different tumor models in order to capture spatial localization of the [2,3-^2^H_2_] fumarate to [2,3-^2^H_2_] malate conversion, before and after treatment. Significant [2,3-^2^H_2_] malate production was shown 48 h following treatment whereas no [2,3-^2^H_2_] malate signals were detected before therapy (Figure 2). Moreover, DMI showed greater sensitivity for detecting cell death compared to hyperpolarized ^13^C, a finding the authors attributed to DMI’s longer time frame in which malate is being accumulated. Taken together, the authors demonstrated effective quantification and spatial resolution of cell death using deuterium MRS, establishing the potential clinical utility of assessing early response to treatment and are currently investigating this probe as an early marker for radiotherapy response in radioresistant GBM tumors [126].

*[methyl-^2^H_3_] methionine*: Methionine is an essential amino acid that has an important role in many cellular functions. In addition to being an essential component in the initiation of protein synthesis, methionine can also take part in (1) the methionine cycle, converted to the important methyl donor s-adenosylmethionine (SAM), (2) polyamine synthesis, (3) folate metabolism, and (4) glutathione synthesis via the trans-sulfuration pathway [127,128]. London et al. administered L-[methyl-^2^H_3_] methionine in excess to rodents in order to investigate its hepatic metabolism in particular its conversion to [methyl-^2^H_3_] sarcosine, clearance of L-[methyl-^2^H_3_] methionine, formation of N-trimethyl-labeled metabolites, and enrichment of water with deuterium [129]. Next, they examined D-[methyl-^2^H_3_] methionine in a similar manner and showed similar metabolism of the agent that can be explained by the rapid transformation of D-[methyl-^2^H_3_] methionine to L-[methyl-^2^H_3_] methionine. This finding was further reinforced when the investigators examined the difference between the L and D enantiomers following inhibition by sodium benzoate, an inhibitor of D-amino acid oxidase, demonstrating a significantly faster formation of [methyl-^2^H_3_] sarcosine following administration of the L enantiomer. However, both enantiomers had markedly reduced rates of methionine clearance and formation of deuterated water, suggesting that sodium benzoate could have an effect on methyl group metabolism in the liver in addition to its effect on D-amino acids [130].

## 5. Bacteria-Specific DMI

**Figure 3:**
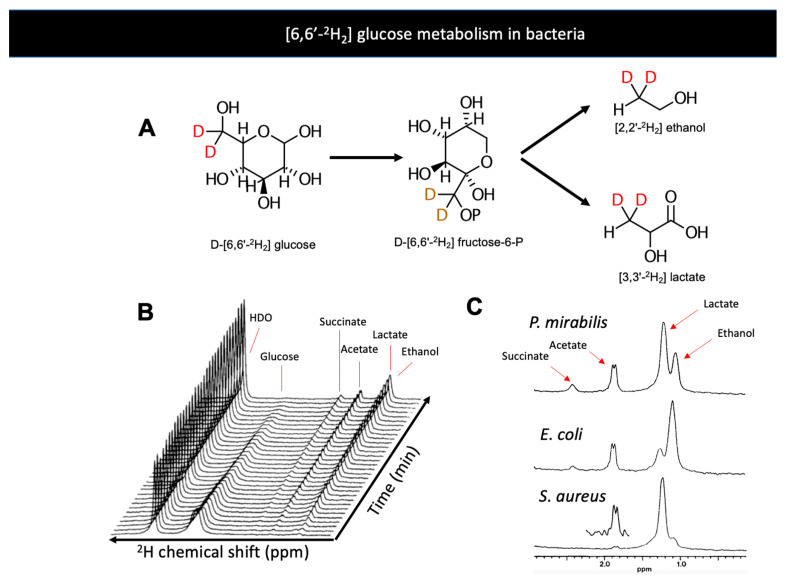
(**A**) Bacterial metabolism of D-[6,6′-^2^H_2_] glucose produces the metabolites D-[6,6′-^2^H_2_] lactate and D-[6,6′-^2^H_2_] ethanol. (**B**) Dynamic conversion of D- [6,6′-^2^H_2_] glucose to metabolic products studied in vitro by ^2^H NMR (Aguayo et al. 1988). The majority of ^2^H signals are incorporated into lactate and ethanol over time (just downfield of 1 ppm). (**C**) ^2^H spectra of products of glycolysis by *E. coli*, *P. mirabilis*, and *S. aureus*, three significant pathogens in humans. Adapted from Aguayo et al. 2021 (Creative Commons Attribution License, CC BY).

The development of metabolic tools that allow detections of bacteria-specific metabolism in a specific and noninvasive fashion has been an ongoing interest for many research groups. In the last decade, several agents have been developed that explicitly target bacteria-specific metabolic pathways, especially using clinically translatable nuclear imaging technologies such as PET [42]. Magnetic resonance studies targeting bacteria-specific metabolism have been infrequently reported. Although MRI is an important modality in imaging infection, there are very few approaches targeting bacterial metabolism. Several manuscripts report using ^1^H MRS to identify the presence of abundant steady-state metabolites in infected tissues, especially in cerebral abscesses [131,132,133,134]. Deuterium magnetic resonance has been previously applied to the study of bacteria via administration of D-[6,6′-^2^H_2_] glucose and identification of the unique bacterial produce [87]. When D-[6,6′-^2^H_2_] glucose was applied to exponential-phase bacterial cultures (*C. perfringens, E. coli, P. mirabilis, S. aureus, K. pneumoniae*), several products were observed that would not be expected in mammalian cells namely acetate, acetone, ethanol, and butyrate (Figure 3). This study also employed the deuterated analogs D-[1-^2^H] glucose and D-[2-^2^H] glucose to show the increased concentrations of HDO that occur with fermentation via the phosphogluconate pathway (C1 enrichment) or glucose-phosphate isomerase (C2 enrichment). These data highlight the way that the metabolism of deuterated substrates can also be detected via the “loss” of ^2^H to water.

## 6. Conclusions

The interest in deuterium imaging has been sparked in the past decade, due to the growing need for non-invasive and non-radioactive imaging methods that are disease specific. DMI showed a remarkable ability to depict metabolism of a ^2^H labeled substrate in three dimensions, allowing the investigation of multiple metabolic processes in both healthy and diseased states, such as infection, malignancy, impaired tissue perfusion and many others. This innovation is therefore considered to have a vast potential, as more and more groups investigate DMI, providing great promise to the future of disease-specific imaging.

**Table 1:** Deuterium MRS/DMI studies in living systems.

Probe	Measured Metabolic Processes	Imaging Target *	Applications	Human Studies	Refs
D-[6,6′-^2^H_2_] glucose	Glycolysis, oxidative phosphorylation, glycogen liver storage	Mammalian cells: [3,3′-^2^H_2_] lactate [4,4′-^2^H_2_] glutamate [4-^2^H] glutamate [4,4′-^2^H_2_] glutamine [4-^2^H] glutamine	Infection, Malignancy, Perfusion	Glioblastoma, Glycogen storage	[1,3,47,71,74,86,87,89,91,92,93,96,97]
		Bacterial: [3,3′-^2^H_2_] lactate [2,2′-^2^H_2_] acetate [2,2′-^2^H_2_] ethanol [2,2′-^2^H_2_] succinate [1,1′-^2^H_2_] 2,4-butaneiol ^2^H-butyrate ^2^H-butanol ^2^H-acetone			
D_2_O	Blood flow and tissue perfusion, DNA and lipid turnovers	D_2_O tracer kinetics Deuterium-enriched DNA	Perfusion, cellular proliferation, lipid metabolism		[75,76,78,99,104,105,106,107,108,109,110,111,112,113,116]
		Deuterium-enriched DNA			
		Deuterium-enriched lipids			
[^2^H_3_] acetate	TCA cycle flux, fatty acid oxidation	[4,4′-^2^H_2_] glutamate	Malignancy	Glioblastoma patients	[1,71]
		[4-^2^H] glutamate			
		[4,4′-^2^H_2_] glutamine			
		[4-^2^H] glutamine			
[^2^H_9_] choline chloride	Phosphorylation, methylation	[methyl-^2^H_9_] betaine	Renal metabolism, Malignancy	Choline deficiency	[119,120,122]
[2,3-^2^H_2_] fumarate	Cellular necrosis	[2,3-^2^H_2_] malate	Malignancy		[123]
[methyl-^2^H_3_] methionine	Protein synthesis, methylation	[methyl-^2^H_3_] sarcosine	Hepatic metabolism		[129,130]
		N-trimethyl labeled metabolites			

* In addition to HDO.

## Data Availability

All data is provided in manuscript.
